# Design, Manufacturing and Test of CFRP Front Hood Concepts for a Light-Weight Vehicle

**DOI:** 10.3390/polym13091374

**Published:** 2021-04-22

**Authors:** Paul Bere, Mircea Dudescu, Călin Neamțu, Cătălin Cocian

**Affiliations:** 1 Department of Manufacturing Engineering, Faculty of Machine Building, Technical University of Cluj-Napoca, Memorandumului 28, 400114 Cluj-Napoca, Romania; 2 Department of Mechanical Engineering, Faculty of Automotive, Mechatronics and Mechanical Engineering, Technical University of Cluj-Napoca, Memorandumului 28, 400114 Cluj-Napoca, Romania; 3 Department of Design Engineering and Robotics, Faculty of Machine Building, Technical University of Cluj-Napoca, Memorandumului 28, 400114 Cluj-Napoca, Romania; 4 SC. BELCO AVIA SRL, Cruci Str. 423, 427120 Livezile, Bistriţa-Năsăud, Romania; cocian.catalin@belcoavia.ro

**Keywords:** carbon fiber reinforced polymers, front hood, manufacturing technology, numerical and experimental analysis

## Abstract

Composite materials are very often used in the manufacture of lightweight parts in the automotive industry, manufacturing of cost-efficient elements implies proper technology combined with a structural optimization of the material structure. The paper presents the manufacturing process, experimental and numerical analyses of the mechanical behavior for two composite hoods with different design concepts and material layouts as body components of a small electric vehicle. The first model follows the black metal design and the second one is based on the composite design concept. Manufacturing steps and full details regarding the fabrication process are delivered in the paper. Static stiffness and strain values for lateral, longitudinal and torsional loading cases were investigated. The first composite hood is 254 times lighter than a similar steel hood and the second hood concept is 22% lighter than the first one. The improvement in terms of lateral stiffness for composite hoods about a similar steel hood is for the black metal design concept about 80% and 157% for the hood with a sandwich structure and modified backside frame. Transversal stiffness is few times higher for both composite hoods while the torsional stiffness has an increase of 62% compared to a similar steel hood.

## 1. Introduction

Reducing vehicle weight is one of the main methods for low fuel consumption in the case of cars that have conventional engines or vehicle autonomy in the case of electrical vehicles. Lightweight is advantageous for maximizing the engine efficiency, accelerating force and braking power in comparison with a heavier vehicle. Automobile manufacturers are seeking to achieve a lightweight structure by structural design and inclusion in the manufacturing process of proper materials such as composite materials. The car producers started considering replacing the doors and bonnets with light-weight structures made of carbon fiber reinforced polymer (CFRP). The Ford Company presented in 2012 to the Reinforced Plastics Journal [1] a CFRP bonnet that weighs 50% less than a steel version. The process for fast and affordable production of automotive parts made of composite materials in a large number is still a challenge, manufacturing of cost-efficient elements implies a proper technology combined with a structural optimization of the material structure. Carbon fiber reinforced plastics (CFRP) components such as a hood should meet the high standards for stiffness, dent resistance and crash performance. The component must also perform well in pedestrian protection head-impact tests. The mechanical properties of CFRP are studied by different authors over time. Tensile strength of CFRP presented in [2,3,4], bending and Charpy impact fracture energy [5,6,7], used different stacking sequences of the layers, indicating very good mechanical properties and lightweight of CFRP structures. The technologies to obtain the CFRP parts are very important, in order to obtain very high mechanical properties of parts and a very compact structure [8]. We can say that, for the moment, the vacuum bag technology and autoclave curing process is the best procedure to produce a very high-quality level of CFRP. A wide range of experimental investigations of CFRP obtained in the autoclave curing process, the authors presented in [9] the importance of autoclave curing parameters and polymerization mechanism. Switching metal parts design to CRFP requires a new product design. Many designers traded the fiber reinforced polymer (FRP) like metal or plastic materials. They designed pieces that copy the metal parts, by reproducing the same geometry shape, the reinforced ribs, all like a quasi-isotropic material approach. The concept of black metal design (BMD) for composites limits the benefits that composites structures have. The arrangement of the fibers in the direction of maximum demands, use of sandwich structures to achieve a high rigidity and light structure, used a balanced stacking sequence of layers in order to respond to complex requests are a couple of the advantages of FRP design concept. The development of a new 3D reinforcement material type [10] or sandwich structures with different types of light cores like Nomex [11,12,13,14,15,16], Aluminum [17,18] or PP Honeycomb, polymethacrylimide closed-cell foam Rohacell or Balsa wood [19,20] contributes to the development of complex light and rigid structures. Sandwich structures with CFRP face sheet and different light cores are experimentally studied in [21]. Proposed in the lightweight body structures in electric vehicles, these structures were analyzed under static and dynamic loading.

In order to replace the steel with new light-weight material in the manufacturing process of a vehicle hood it is necessary to estimate beside the mechanical proprieties of the materials also the structural performance of the same part. The structural performance of the car hood presumes stiffness, modal and pedestrian protection properties. The stiffness of a hood is described by the relationship between load and deformation and it can be evaluated by measurement of flexural displacement produced by an external load. Depending on the application point of the load and supporting conditions, bending and torsion stiffness can be evaluated. Generally, modal behavior can be characterized by 1st torsion mode and bending mode. Property of pedestrian protection for hood usually consider the associated collision pedestrian protection standards, the Head Injury Criterion (HIC) being an important parameter to evaluate the pedestrian protection level. The outer hood panel is shaped according to the vehicle design and it cannot be subjected to any structural improvements excepting the layer’s architecture in the case of composite materials. The inner panel of the hood can be structurally optimized in order to improve the stiffness, modal behavior and pedestrian protection both by topological and material design without changing the clearance between the inner panel and engine zone. Related studies have shown that the structure of the inner panel of the hood has a greater impact on stiffness [22] and impact behavior [23,24]. Numerical and experimental investigations of aluminum car hood presented in [25] indicate that the optimized hood structure shows similar stiffness performance compared to the original steel structure and better pedestrian protection performance than the original ones; the proposed aluminum structure realized a weight reduction of 46.4% compared with the original steel one. Composite materials were considered as an alternative to steel and aluminum for hood structures. The main manufacturing steps of a CFRP front hood and a proposal for the composite material layers’ distribution are presented in [26]. The inner panel design has a hexagonal shape reinforcement. Vacuum-as resin transfer mold (VRTM) was employed to obtain the CFRP front hood. The composite material was a balanced woven fabric Twill 2 × 2 by 200 g/m^2^ with the distribution of layers [90/0 ± 45_2_/90/0]. The analysis of the typical load cases and computation of lateral, transversal and torsional stiffness of the front hood was done using finite element commercial software and proved that the proposed design of the composite material and hood structure can fulfil the requirements of the standard static tests. A similar study but different composite material layup is presented in [22], the proposed hood has no inner part, the outer surface is designed to replace the inner part by a composite material with different mechanical proprieties. The behavior of three identical bonnets made of steel, aluminum and composite have been investigated in [23] in terms of head impact. The developed composite model (5-ply Glass/Epoxy) showed excellent results in HICs and torsional stiffness besides being lightweight, although it has the highest cost among the bonnets. Numerical modelling of a hood made of CFRP composite are presented in [24]; the work concluded that using composite material for the hood structure there was a reduction in HIC values and an increase of the head displacement compared to other materials. In [27], an automobile bonnet manufactured of flax/vinyl ester composite that is being researched in various ways as eco-friendly material was evaluated to perform structural design and analysis. All hoods’ variants made of composite materials must satisfy bending and torsion stiffness requirements compared to those of conventional steel and aluminum hoods.

However, very few studies on the hoods presents a complete picture of the static behavior of a CRFP hood and proposes specific design, complete manufacturing detail, experimental investigations and numerical studies. This paper presents the mechanical behavior of a CFRP hood manufactured considering two different solutions. One solution where the inner part mimics the original steel bonnet BMD concept and the second one that replaces the ribs and the reinforcements with a sandwich architecture. In the first part of the paper, the geometrical topologies of the investigated hoods, CFRP materials employed for hood design and the elastic material constants experimentally determined on flat specimens according to standard procedures are presented. The second part is dedicated to manufacture technology of hoods and presents detailed aspects regarding the process steps, parameters and methodology. A 3D scanning technique revealed the manufacturing dimensional accuracy with respect to CAD models. The fabricated hoods are experimentally tested using a self-developed testing frame under similar boundary condition as in reality. Static stiffness for longitudinal, transversal and torsional load cases are evaluated by measuring the displacement produces by a concentrated force acting perpendicular to the hood upper surface under different supporting conditions. Strain values in three different points placed on upper and lower hood’s surfaces are monitoring as the load is applied to get information about the strain state in the structure of the hood. Comparison of the mechanical behavior of the experimental hoods with numerical models was performed by finite element analyses. Good agreement between displacements and strains validates the numerical analyses and will support future static and dynamic analyses.

## 2. Materials and Methods

### 2.1. Hood Design Concepts

**Figure 1:**
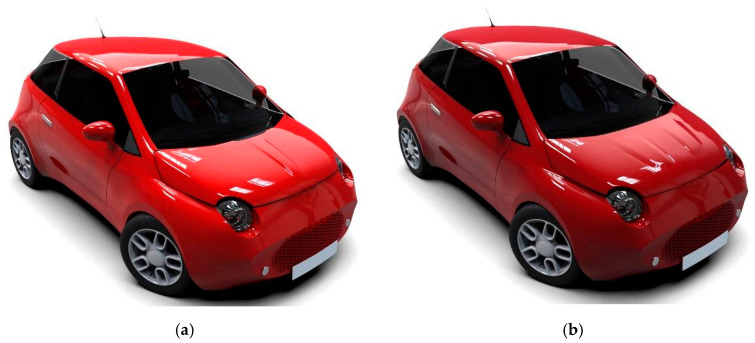
The concept of the EV: (**a**) front hood “A” design, (**b**) front hood “B” design.

The development of the novel electric vehicle (EV) with small dimensions led to the design and manufacturing of components as light as possible. Figure 1 presents rendered models (Catia V5 software) of the developed EV concept. The main purpose of the new EV concept is to reduce the mass and increase the range. In this case, CFRP body-in-white parts were chosen to replace traditional materials such as conventional steel.

For this study, two front hoods from CFRP were designed, analyzed and manufactured. In the first step was chosen a BMD concept based on a replica of a similar metal front hood but with the new material. In our case, the hood is made of CFRP prepreg using different materials and stacking sequence for the layers. The first version of the hood, denoted “A”, made according to the BMD concept has two main parts an exterior face and a backside frame. The backside frame includes all the interest points of the hood such as support points on the chassis, hinge fastening points and closing area. After manufacturing an experimental and numerical study was performed to evaluate the mechanical behavior under static loads in terms of longitudinal, lateral and torsional load cases. A second variant, denoted “B”, is an optimized composite design and shape of the backside frame. The aesthetic differences between the hoods are minor on the outside face, the initial design has a single rib (Figure 1a) on the middle and the second one has been slightly changed by inserting two less pronounced ribs on the central side (Figure 1b).

For the second hood, the optimized model includes changes in the CFRP structure too and the backside frame dimensions were reduced to decrease the mass of the hood. Experimental and numerical analyses were performed on the second variant to compare the results in terms of stiffness with the first variant and a metal (steel) hood. The studies are complex including material selection, material testing and characterization, manufacturing of the front hoods at real scale, static tests under different loads and boundary conditions and numerical simulations.

### 2.2. Materials and Mechanical Proprieties

In the previous studies of [22,26], the proposed CFRP layup was a balanced woven fabrics Twill 2 × 2 (200 g/m^2^) with the distribution of layers [90/0 ± 45_2_/90/0] or more complex structure including a Twill fabric (200 g/m^2^) combined with a biaxial fabric of 300 g/m^2^ and a Nomex honeycomb core. Based on the numerical results obtained and expertise gained by the manufacturing process of one of these hoods, in the present paper, two new and different design variants of the composite hood are presented. For an easier description, the two variants are referred to as variant “A” and “B”, respectively.

**Figure 2:**
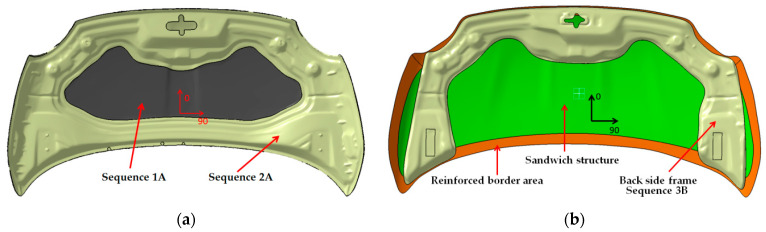
The CAD model of the backside of hoods. The positioning of the CFRP sequences on hood: (**a**) front hood variant “A”; (**b**) front hood variant “B”.

For the “A” hood made by BMD concept, the following materials and stacking sequence of the layers are presented in Figure 2a. The sequence 1A consists of three layers of CFRP prepreg. The first layer was CFRP type GG245TSE-DT121H-42. Reinforced materials were 2 × 2 Twill fabric, 245 g/m^2^, 2 K, HR threads and the next 2–3 layers CFRP prepreg type GG430TSE-DT121H-42. In this case the reinforced materials were 2 × 2 Twill fabric, 430 g/m^2^, 12 K, HR threads. The stacking sequence of the CFRP for “1A” was noted as [90/0 ± 45/90/0].

For the sequence “2A” which represented the backside frame of the hood two layers from the same materials were used. The first layer applied to the mold was CFRP prepreg type GG245TSE-DT121H-42 and the second one GG430TSE-DT121H-42 type. The stacking sequence of the layers was [0/90/ ± 45]. The position of the layers was in the order of laminating on the mold. All the mentioned materials were provided from Delta Tech S.p.A. Company from Rifoglieto Italy.

The second studied hood noted “B” was manufactured using CFRP prepregs. The structure of this hood was different from the hood “A”, in this case being used a sandwich structure. The purpose of this new concept (presented in Figure 2b) is to reduce the mass of the hood and achieve a good mechanical response of the structure. This concept removed a large number of carbon fibers. For the backside frame, the points of interest were preserved and a minimal backside frame was introduced around the interest points.

**Figure 3:**
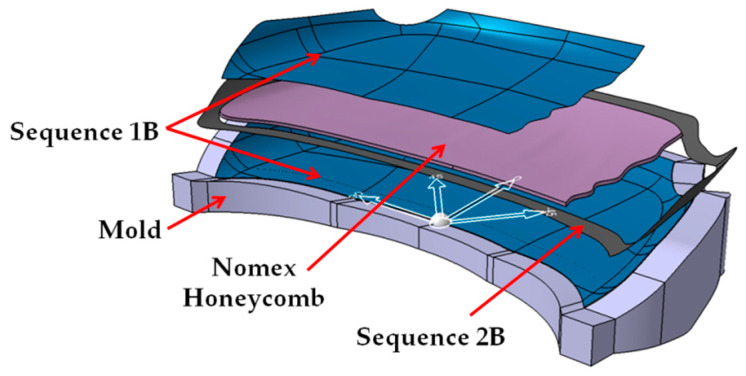
The stacking sequence of layers for the front hood: B. exterior surface.

The details regarding the stacking sequence of the applied layers for “B” hood are presented in Figure 3. The sequence 1B represented the exterior layers of the sandwich structure of the hood. Three CFRP prepreg layers GG090P-DT121H-48 type were used and combined with a plain fabric, 90 g/m^2^, 1 K, HR threads type of CFRP as reinforced material. The stacking sequence was [0/90/ ± 45/0/90]. For the interior the material is a Nomex honeycomb by 10 mm and 3.2 mm size with hexagonal cells.

The sequence denoted by 2B is positioned around the edges of the hood and uses three layers of Biaxial CFRP prepreg with [±45]_3_ stacking sequence. The width of the applied strips was 50 mm and the selected material was G300X(T700)-DT121H-37 a CFRP biaxial fabric, with 300 g/sqm as reinforced material. In the border of the hood, the sandwich structure was not applied. All these layers were covered by a 1B sequence. Both the Nomex structure and the 2B sequence were covered. For the backside frame of the “B” hood (sequence 3B presented in Figure 2b), four layers of CFRP prepreg GG245TSE-DT121H-42 type were used. The stacking sequence was [0/90/ ± 45]_2s_.

**Figure 4:**
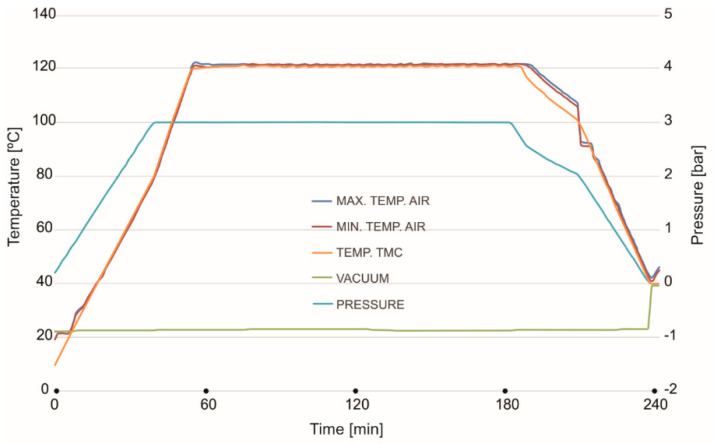
Autoclave curing cycle diagram.

Before hoods manufacturing, a mechanical characterization and measurement of elastic constant of component the materials are necessary. In this sense, several plates having the material layups above described have been fabricated. The manufacturing technology of the proposed CFRP plates was vacuum bag technology and autoclave curing process. For manufacturing of the CFRP plates, the flat metal mold was used. The surface of the mold has been rectified and polished. On the active surface of the mold, mold release liquid was applied The CFRP prepreg layers were laminated on flat mold. In the end, the CFRP layers were covered by release foil and the breeder. The entire system (mold and laminated prepreg composites) was introduced in a vacuum bag, closed at the edges and applied a vacuum pressure at −0.9 Bars. The curing process of CFRP plates was carried out in an autoclave. The cycle steps and parameters set are presented in Figure 4 and include: Step 1. Autoclave heated from 20–80 °C at 2 °C/min ramp rate for 30 min; pressure from 1–3 Bars. Step 2. Autoclave heated from 80–120 °C at 2 °C/min. ramp rate for 30 min; pressure 3 Bars. Step 3. Dwell at 120 °C for 120 min; pressure 3 Bars, vacuum pressure −0.9 Bars. Step 4. Cool the part from 120–50 °C at 2 °C/min for 60 min, eliminate the pressure. In all cycle steps the Vacuum pressure was −0.9 Bars.

The materials, parameters and conditions are similar to those used for the manufacturing of the hoods. To perform the experimental determinations standard specimens were cut by water jet from the CFRP obtained plates. Tensile tests following the ASTM D3039M standard were run for specimens with the warp fibers parallel to the load. Uniaxial tensile test of a ±45° laminate is performing by the ASTM D3518M standard to evaluate the in-plane shear response. Elastic modulus, shear modulus and Poisson’s ratio have been derived from these tests. In this study, a homogenization procedure is implemented; the material model adopted for numerical simulations is an isotropic elastic model.

For accurate measurements unidirectional (1-LY1x-6/120) and bidirectional strain gauges (1-XY91-6/120, HBM, Darmstadt, Germany) with 120 Ω electrical resistance and 6 mm gauge length were applied to each specimen to monitor the longitudinal and transverse strain [3]. The bidirectional strain gauges have two overlapped grids able to measure the strains in the same point in two directions. A half-bridge set-up with passive strain gauges mounted on a non-loaded specimen of the same composite material was used to compensate for the temperature variation. The tests for elastic constants identification were conducted on an INSTRON 3366 (10 kN) universal test frame controlled by an electronic control unit which allows monitoring of the applied load and the speed of the crosshead. Strain signals and a second load cell were acquired by a digital data acquisition system (HBM Spider 8, Darmstadt, Germany) [3].

In Table 1 and Table 2 are given the obtained results from the tensile test of specimens having the stacking architecture and materials described in the previous paragraph. The values represent the mean values of several experiments.

**Figure 5:**
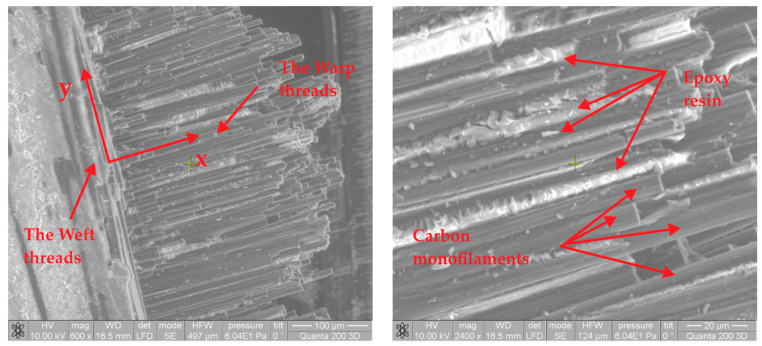
Microstructure analyses of CFRP samples subjected buy tensile test.

To analyze the tested CFRP microstructure, a morphological study was performed. The fracture areas of the CFRP tested specimen were analyzed using Scanning Electron Microscopy (SEM) and a Quanta 200 3DDUAL BEAM SEM type (FEI Company, Hillsboro, OR, USA). Each of this CFRP segments are assembled in an aluminum support specific for SEM analyses of a microstructure. The Low Vacuum module offer the possibility to analyze the natural surfaces of CFRP samples, thus covering the samples surface with an electrically conductive coating was not necessary. Working parameters were set to 60 Pa for the working pressure and 10 kV for acceleration voltage. This relatively low voltage prevents electrostatic charging of the samples. The detector type was large field detector (LFD). To clearly observe the architecture of the surface in the fracture area and the CFRP structure the images were acquired at higher magnification (600×, 2400×). Microstructures of CFRP plates subjected to tensile tests indicate a very pressed material. The pores in the material structure are not present, the monofilaments are closed and the epoxy matrix is uniformly distributed (Figure 5). This binds the monofilaments which act together in case of traction.

Parts of the epoxy resin left on the surface of the carbon monofilaments can be also observed and indicates a very good cohesion between carbon monofilaments and the epoxy resin. Microstructure investigation of analyzed samples in the fracture area presents a high quality CFRP composite with a good resin impregnation of monofilaments and no delamination.

### 2.3. Manufacturing Methodology of CFRP Hoods

Molds were used for manufacturing the hoods both for the front exterior side and for the back frame side. The milling CNC Epoxy block was used for fabricating the molds. The obtained surface of milled molds was sanded using a glass paper by 400–1500 grit. The surface molds were treated at the end using an abrasive polishing paste. In order to prevent the sticking of CFRP prepreg on the mold surface a release treatment procedure was applied. The procedure consisted in applying of 4 layers of Mold Sealer type S31 from Jost Chemicals Company (Wals, Austria). This substance closed the surface pores of the mold. In the next step were applied 5 layers of liquid mold release type Frekote 770NC from Loctite Company, 10 min wait period after each layer was needed in order to allow drying of the solvent. The end step in mold preparation was surface polishing.

The manufacturing of the CFRP hoods were made using vacuum bag technology and autoclave curing. For the “A” hood, the CFRP prepreg layers were laminated on the mold according to stacking sequence above presented. On the backside, the edge of the hood a peel ply layer was applied to cover the bonding area of the back frame. Release foil, textile breeder and vacuum foil covered the CFRP Prepreg layers at the final lamination. Mastic tape was used for sealing the vacuum foil on the edges of the molds. In the end, the vacuum bag was tested one hour under −0.9 Bars vacuum pressure for 30 min.

The CFRP laminates and the molds were introduced in the autoclave for curing procedure. The same cycle steps and the parameters were used the same as they were used in the manufacturing of CFRP plates described in the previous paragraph. For the backside frame were respected the same curing condition of the manufacturing procedure as for the exterior part. The backside frames and the exterior side of the hood were bonded using a structural adhesive Scotch Weld, BP-9323 B/A type from 3M Company (Bracknell, UK). In order to eliminate the possible deformations of the parts, the gluing was done in the mold.

The curing procedure of the structural adhesive at 80 °C run for two hours. The material excess from the borders of the hood were eliminated at the end using a manually mechanical procedure.

**Figure 6:**
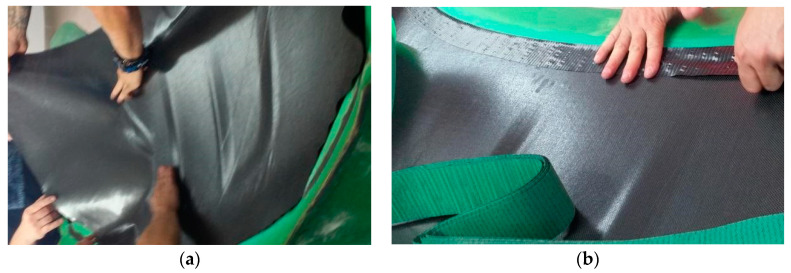
CFRP laminating process on hood mold: (**a**) laminating of CFRP surface layer; (**b**) laminating of reinforced borders with CFRP biaxial fabric.

For the second front hood marked with “B” the same mold was used. The first three sequence layers of P90 CFRP were applied to the mold (Figure 6a). On the borders of the hood, three layers of Biaxial CFRP with 300 g/m^2^ were applied together with a peel ply textile layer. The result was a 50 mm reinforced area around the borders of the hood (Figure 6b).

**Figure 7:**
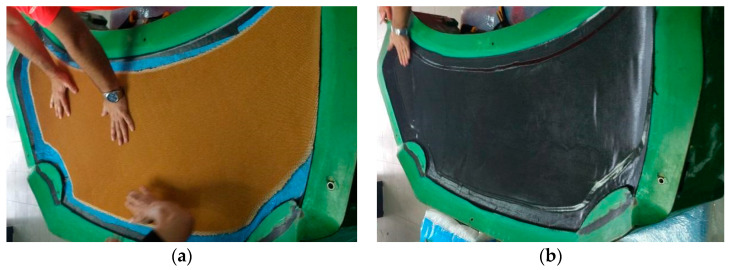
Sandwich structure manufacturing: (**a**) Nomex Honeycomb applied on CRFP layers; (**b**) covering of Nomex Honeycomb with CFRP prepreg.

All the applied CFRP layers were cured in an autoclave as previously mentioned. After the curing procedure of the exterior side of the hood, the Peel ply was eliminated. The obtained surface was covered by a prepreg adhesive film type AX003-150-30-6000F. This allows the connection between CFRP layers and the honeycomb structure (Figure 7a). The Nomex honeycomb structure was cut by an offset of 50 mm than the outer edges of the hood. The Nomex structure is present starting from the backside of the hood until biaxial CFRP reinforcement. The last three CFRP layers of P90 (sequence 1B) covered the entire structure (Figure 7b). The curing procedure was done in autoclave condition. The parameters of the autoclave remained the same excepting the curing pressure reduced to at 1.5 Bars during all steps of the autoclave cycle to avoid damaging of the honeycomb structure. The backside frame of the hood was manufactured using the same conditions previously presented in the case of hood “A”. The bonding of the backside frame was done using Scotch Weld structural adhesive in similar conditions as for the previous hood.

For the obtained CFRP hoods, the surface structure is very good and surface pores are not presented. The front hood has very good rigidity and the CFRP material is very compact and has a homogenous structure, as we can see in the morphological analyses.

**Figure 8:**
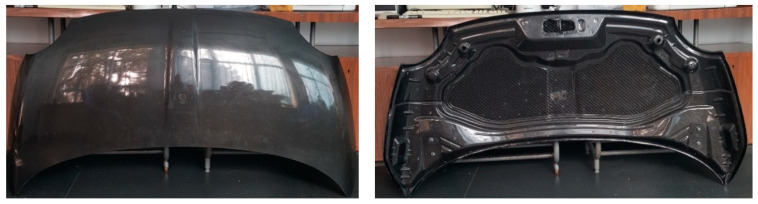
The obtained CFRP front hood “A” made according to the BMD concept.

**Figure 9:**
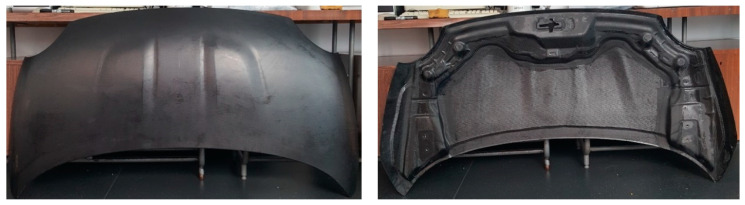
The obtained CFRP front hood “B” made by sandwich structure.

The mass of the “A” front hood (Figure 8) was 3407 g. For the second front hood, “B” (Figure 9) the mass was 2690 g. The mass reduction is 22% in the case of the sandwich structure hood.

For the exterior surface of the hood “B”, which did not include the backside frame, the obtained mass was 1626 g. In this case the mass reduction is 53%. Considering this reduction, the backside frame must be eliminated and for the interest points like support points on the chassis, the hinge fastening points and closing area support must be reinforced. Mass reduction can be between 40–50% in the hood B case. At the same time, we can say that the stiffness of the hood decreases if we eliminate the backside frame.

### 2.4. Dimensional Evaluation of the Obtained CFRP Hoods

After the manufacture of the two hood models, they were dimensionally evaluated. The evaluation was realized to verify how the manufacturing process influenced the precision of the parts. The dimensional evaluation of the CFRP hood was done by scanning, the obtained results were compared with the CAD model of the hoods.

Scanning was performed with a white structured light scanner, with the following specifications: measurement rate—550,000 measurements/s; resolution—0.100 mm; accuracy—0.100 mm (Go!SCAN—Creaform Inc., Lévis, QC, Canada). Scanning was performed with position targets, as recommended by the manufacturer, to increase scanning accuracy. Because the thickness of the piece is relatively small in relation to the length and width, three different scans were performed, one for each face and a partial scan for both faces. During the processing stage, the three scans were concatenated into a single model using the VXElements software solution (Creaform Inc., Lévis, QC, Canada).

**Figure 10:**
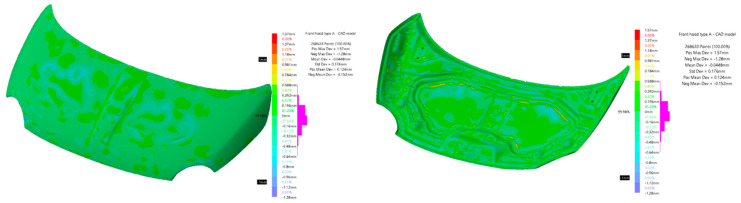
CAD model—scanned model comparison for hood “A”.

The verification is done by comparison between two 3D models using the Deviation Analysis tool from CATIA V5 (Dassault Systèmes—Vélizy-Villacoublay, France). Figure 10 shows the deviation analysis between the CAD model used in the manufacture of molds and the CAD model resulting from the processing of scans made on the hood noted by “A”. In the analysis, 268,633 points were used of which 87.9% are between ±0.2 mm and 99.98% are within ±1 mm. The farthest point scanned from the CAD model is at 1.57 mm, the standard deviation for all geometric deviations of the scanned model from the CAD model is 0.176 mm.

**Figure 11:**
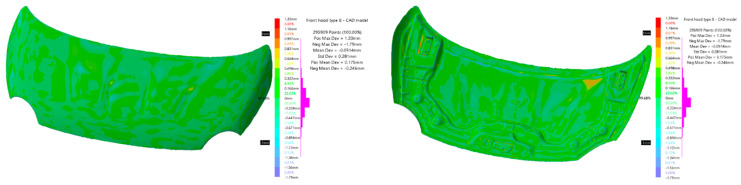
CAD model—scanned model comparison for hood “B”.

Figure 11 shows the deviation analysis between the CAD model used in the manufacture of molds and the CAD model resulting from the processing of scans made for the hood noted “B”. The analysis used 295,909 points of which 83.57% are between ±0.3 mm and 99.68% are within ±1 mm. The farthest point scanned from the CAD model is at 1.79 mm; the standard deviation for all geometric deviations of the scanned model from the CAD model is 0.281 mm.

## 3. Results

### 3.1. Experimental Stiffness Investigation of Composite Hoods

**Figure 12:**
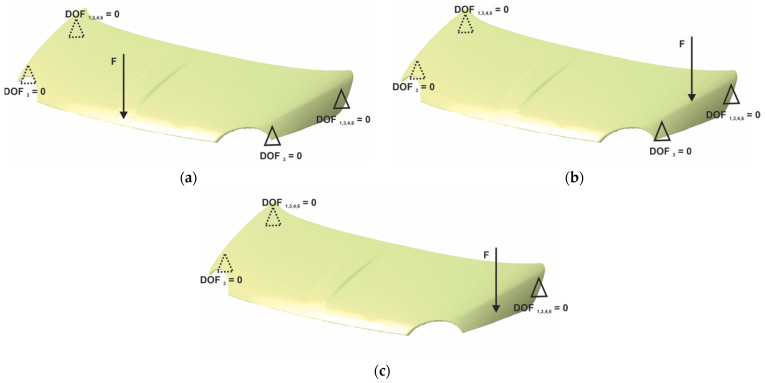
Boundary conditions used for evaluation of: (**a**) lateral stiffness, (**b**) transversal stiffness and (**c**) torsional stiffness.

Hoods structural stiffness evaluation usually consists of three types of loads: transversal bending, longitudinal bending and torsion. The criterion for stiffness tests is the elastic deflection due to the applied load. In all load cases, the hood is mounted in real position being constrained in the supporting points, the hinges in the rear and the buffer points in the front, respectively. The supports are defined for lateral, transversal and torsional stiffness estimation by their degrees of freedom (DOF) from 1 to 6 (1, 2 and 3 stand for translational constraints in *X*, *Y* and *Z* axis, respectively, and 4, 5 and 6 stand for rotational constraint about *X*, *Y* and *Z* axis, respectively) as shown in Figure 12. The orientation of the Cartesian coordinates system considers *X* axis as the longitudinal axis of the car, *Y* axis is oriented in the transversal direction and *Z* is the vertical axis. The loads are applied in the vertical direction and have for each load cases different intensities. A conventional steel hood is used to establish the reference data for the load intensities and the stiffness requirements. Thus, for lateral and longitudinal stiffnesses a concentrated force between 400 and 500 N is employed, while the torsional stiffness considers a force about 100 N [28,29,30,31].

**Figure 13:**
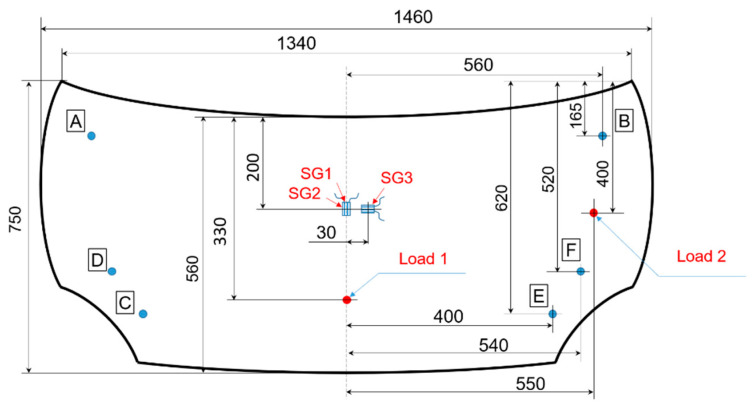
Supports and application points of the loads for investigated composite hoods.

Experimental set-up for stiffness evaluation consists of a rigid frame that ensures supporting conditions and load application. For the manufactured hood, the supporting points are presented in Figure 13 and consists of two hinges (A, B with the restricted DOF 1, 3, 4, 6) and two bumpers on each side (D, C, E, F with the restricted DOF 3). For lateral and transversal stiffness measurement, all supporting points were employed; in the torsional loading case, the E and F supports were removed.

**Figure 14:**
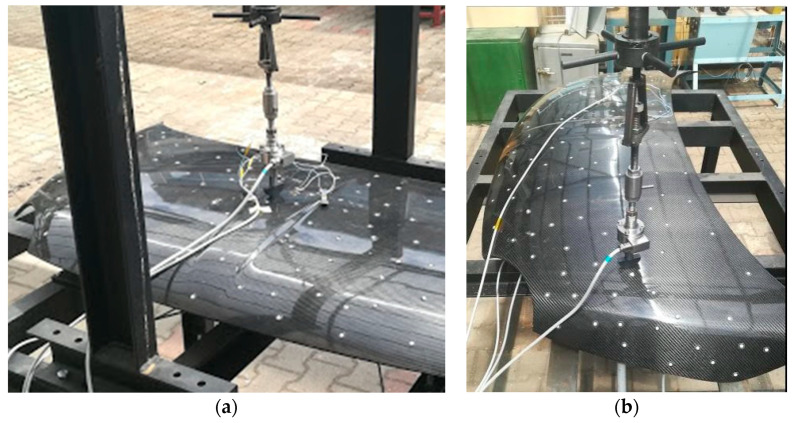
Experimental set-up for structural analyses of the composite hood “A”: (**a**) load position for lateral stiffness evaluation and (**b**) load position for transversal and torsional stiffness.

The frame was instrumented with a simple load application device that converts the rotation of a nut into a linear movement of a screw. A force transducer type HBM F2B 10 kN (HBM, Darmstadt, Germany) connected to an amplifier HBM Spider 8 allows real-time force value acquisition. The deflections due to the load application were measured by linear inductive displacements transducers type HBM WA-T with a 10 mm measurement range fixed on the opposite face of the bonnet colinear with the load. Application points of the forces and displacements transducers are also presented in Figure 13. For additional information about the strain–stress state, several strain gauges were mounted on the hood surface and connected at the same amplifier. One unidirectional strain gauge (SG1) was glued on the outer surface in the marked point (Figure 13) in the longitudinal (x) direction of the hood and the other two strain gauges on the inner surface (SG2 in the longitudinal direction and SG3 in transversal direction (y)). Each strain gauge was connected in a half-bridge circuit with one dummy strain gauge for temperature compensation glued on the unloaded specimen made of similar composite materials. All measurement data (load, displacement and strains) were analyzed by the HBM CatmanEasy data acquisition and measurement software. Instrumented set-up for hood structural analyses is presented in Figure 14.

The hood “A” was loaded progressively with concentrated forces up to 500 N for the lateral and transversal stiffness and 50 N for the case of torsional stiffness, respectively. Several measurements of the load displacements values were recorded, the stiffness value being calculated through linear interpolation. The obtained mean values are presented in Table 3.

Table 4 presents the measured strain values corresponding to maximum force applied to the hood in each loading case, lateral, transversal and torsional.

**Figure 15:**
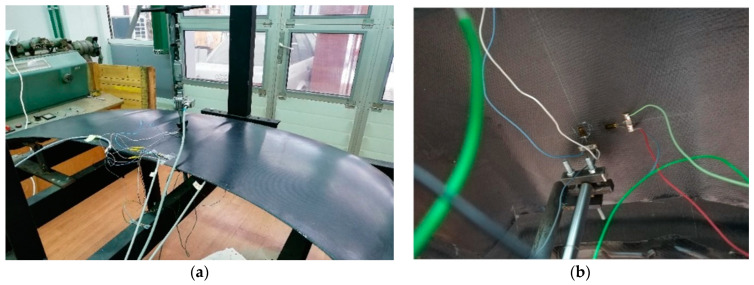
Experimental set-up for structural analyses of the composite hood “B”: (**a**) load position for lateral stiffness evaluation and (**b**) strain gauges applied in longitudinal and transversal direction on the lower face.

Structural analysis of the composite hood manufactured with a sandwich structure (hood “B”) was realized on the same experimental set-up as for the first variant (Figure 15a). Strain gauges were glued in longitudinal and transversal direction of the hood in two points on the upper and lower surface. The position of application points of the loads and strain gauges is presented in Figure 15b. The load for lateral stiffness investigation was about 230 N, for transversal stiffness 524 N and 206 N for the torsional loading case.

Table 5 and Table 6 presents the experimental values of displacements and strains measured for the composite hood “B” with sandwich structure.

### 3.2. Numerical Analyses of Investigated Hoods

The finite element models of the hoods are based on the CAD geometries of the above-described hood, for the simulation being employed ANSYS Workbench 2019 software (ANSYS Inc., Canonsburg, PA, USA) and the ACP tool for layup modelling of the composite materials. The composite material used in the simulation was defined as in the manufacturing process, the isotropic material proprieties of the layers are those experimentally determined or computed numerically in case of a stack-up of more layers. For computational and accuracy reasons, a shell model was chosen to be modelled. The upper and lower hood faces were designed with CATIA software in order to obtain delimited shell surfaces representing the mid surface of the composite material. Surfaces with the same material proprieties were joined and the thickness was defined as symmetrical, top or bottom, with respect to the CAD geometry, in such a way to obtain a numerical model identical to the real one. The connection between the inner and outer surface of the hoods is a bonded connection in the contact areas. The numerical analyses are respective of the established supporting points, their free and cancelled DOFs and application points of loads as presented in the experimental analyses to determine the lateral, transversal and torsional stiffness of the hoods.

For hood “A”, based on the black metal design concept, in the first step was simulated a geometrical identical model having structural steel (E = 200 GPa, G = 76.9 GPa, *ν* = 0.3) as material and a shell thickness of 0.6 mm. The idea is to compare our CFRP hoods with the conventional ones manufactured from steel, a widespread material in the automotive industry. Table 7 presents the stiffness values obtained for the “A” hood made of steel. All boundary conditions and load position are identical to those presented in experimental analyses.

In the case of the hood “A” made of CFRP materials with the lay-up and mechanical constants previously presented, in Figure 1 are shown the boundary conditions and the numerically computed displacements under the load cases and supporting conditions for evaluation of lateral, transversal and torsional stiffness.

**Figure 16:**
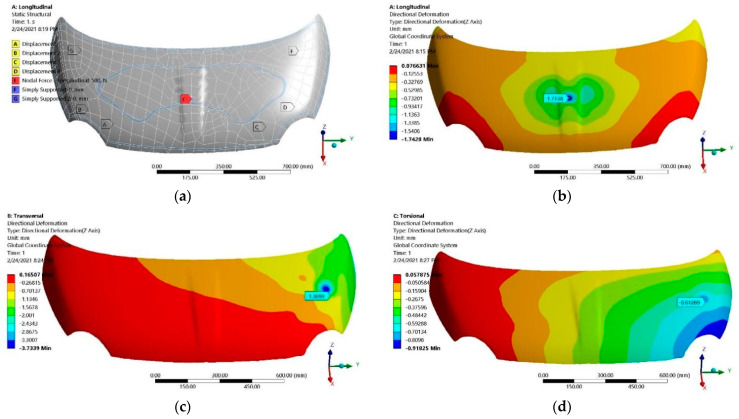
Numerical simulation of hood “A”: (**a**) boundary conditions for longitudinal loading case; (**b**) displacements—longitudinal; (**c**) displacements—transversal; (**d**) displacements—torsional.

Directional displacements were recorded in the load application points that, for the longitudinal load case (Figure 16b), is on the same face (upper part of the hood); for the other cases (transversal and torsional), the corresponding displacement that was experimentally measured on the lower surface of the hood. For similarity reasons, these points and their corresponding displacements are marked in the numerical models and used for corresponding stiffnesses computations. The values extracted for the FE simulations are recorded in Table 8 for the applied forces and their corresponding displacements and in Table 9 for the strain measured in the application points of strain gauges applied on the upper and lower surfaces of experimentally investigated hoods.

Hood “B” was numerically analyzed using the same methodology. In this case, the sandwich structure consists of more plies as described in previous paragraph. A homogenization procedure is necessary to get the elastic constants of shell elements representing the reinforced frame and sandwich structure of the hood (Figure 2b). The materials of these plies were defined in ANSYS Pre-Post Composite software module, resulting in a homogenized material for each zone of the hood “B”. For the honeycomb (Nomex structure), the material constants were given by the producer and are presented in Table 10. After homogenization, the resulting isotropic material constants for the reinforced frame and sandwich structure are presented in Table 11.

**Figure 17:**
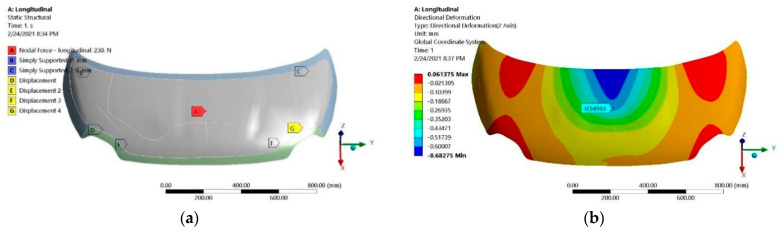

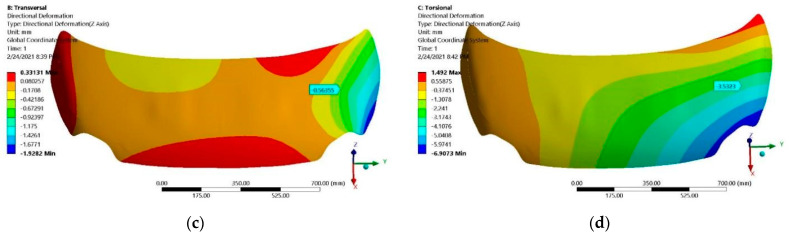
Numerical simulation of hood “B”: (**a**) boundary conditions for longitudinal loading case; (**b**) displacements—longitudinal; (**c**) displacements—transversal; (**d**) displacements—torsional.

Figure 17 presents the boundary conditions of longitudinal load case and the displacements in the application point (longitudinal load case) or in collinear corresponding point on the other face of the hood (for transversal and torsional load case).

The obtained values were used for stiffness calculation. The displacements and strain values extracted for the FE simulations of the hood “B” are presented in Table 12 and Table 13 for the analyzed load cases.

### 3.3. Discussions

**Figure 18:**
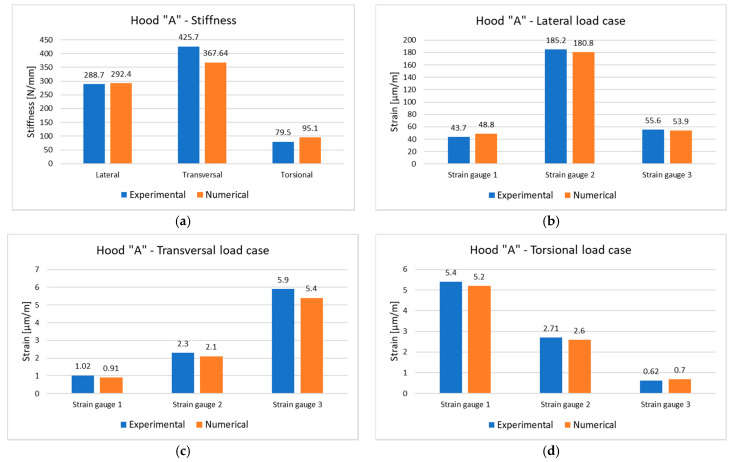
Comparative analysis of experimental and numerical (simulated) values for the composite hood “A”: (**a**) stiffnesses; (**b**) strains in lateral load case; (**c**) strains in transversal load case; (**d**) strains in torsional load case.

**Figure 19:**
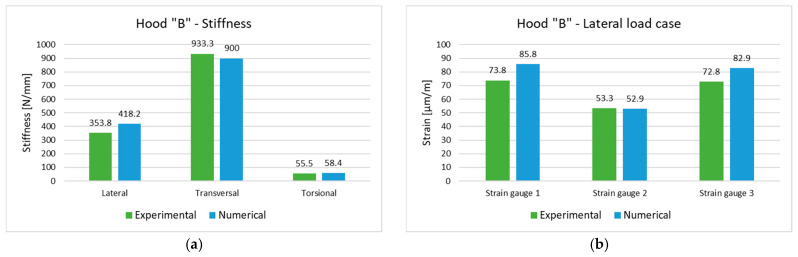

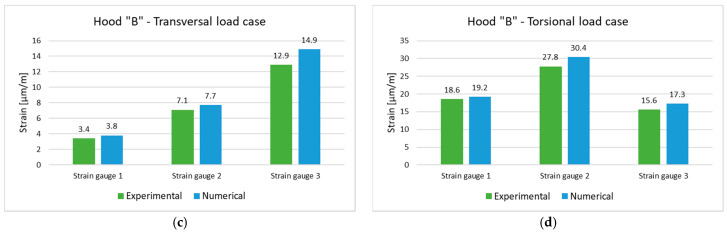
Comparative analysis of experimental and numerical (simulated) values for the composite hood “B”: (**a**) stiffnesses; (**b**) strains in lateral load case; (**c**) strains in transversal load case; (**d**) strains in torsional load case.

Comparative analysis of the experimental and numerical results is focused on stiffness values and the strain registered at the measuring points above presented. A good agreement between the experimental measurement and numerical (finite element) simulations is presented in Figure 18 and Figure 19. In general, the relative deviation between experimental and numerical values in terms of displacements, strains and stiffnesses are under 10%; in few cases, the difference is larger and is due mainly to imperfect experimental tests (supporting conditions, application point of load or application point of displacement transducer). The obtained results validate both the experimental set-up and numerical models of the two investigated hoods.

The strain results for the hood “A” show significant values in case of lateral load case comparatively with transversal and torsional cases for all strain gauges, due to the proximity and position of the application point of the load. In the case of the hood “B”, the behavior is slightly different mostly for transversal and torsional load cases where the generated strain is higher than for the first variant of the composite hood. The effect relates to the material structure and with the backside frame that is no longer continuous over the hood and changes the mechanical behavior under transversal and torsional loads.

**Figure 20:**
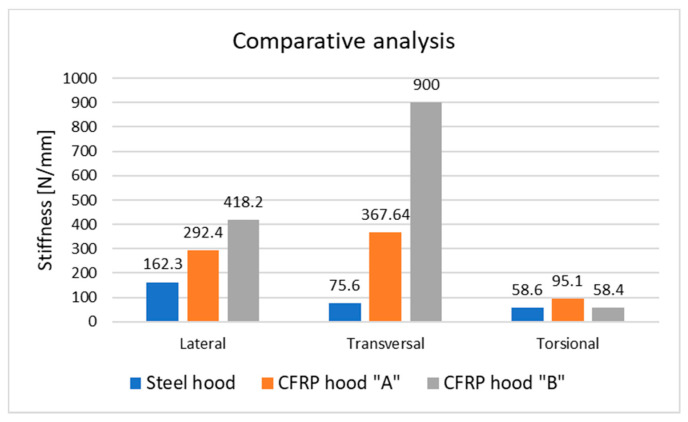
Comparative analysis of the stiffness of investigated hoods.

Both composite hoods show a higher transversal stiffness than lateral and torsional ones, mainly due to their design with an under-unity ratio between longitudinal and transversal dimensions and the presence of two supporting points on each side near the headlamps. Figure 20 represents a comparative analysis between the stiffness of a steel hood, a composite hood with similar design and a composite hood with changed design of the backside frame. It can be noticed that composite hoods are offering superior stiffness to a similar steel hood.

The total mass reduction is 22% in the case of the hood “B” having the sandwich structure and modified backside frame related to BMD structure (“A”). For the exterior surface of the hood “B” which not include the backside frame the obtained mass was reduced by about 53%. The computed mass of the steel hood with the same geometry as in the BMD concept indicates a value of 6856 g. This mass was determined according to the CAD model of hood “A” using CATIA V5 software. Compared with the steel hood, CFRP hood “A” has a two-fold mass reduction and hood “B”, made by sandwich structure and CFRP faces, has a mass reduction of 254 times. If we eliminate the backside frame of the hood, “B” de mass reduction is about 421-fold. This difference is remarkable if we look at the results presented in Figure 20 one can notice that the stiffness of CFRP hoods is higher than the steel one and hood “B” the lightest structure has the highest lateral and transversal stiffness compared with all other hoods. In the torsional stiffness, the obtained results are comparable. The improvement in terms of lateral stiffness for composite hoods about a similar steel hood is for the hood with black metal design about 80% and 157% for the hood with sandwich structure and modified backside frame. Transversal stiffness is significantly higher for composite hoods, three times higher for hood “A” and 12 times for hood “B”. The hood designed as a sandwich structure is transversally stiffer (245 times) comparative with the hood made by BMD concept. Torsional stiffness is less influenced by changing of materials in case of hood “A” is an increase of 62% and a similar value as for the steel hood is obtained by composite hood “B”. This can be explained by similar backside frame geometry in the case of the first composite hood, but different material and in the second composite hood by changed design of this back frame.

## 4. Conclusions

The paper presents a complex study regarding two CFRP front hoods for small EV. A very few studies on the hoods presents a complete picture of static behavior of a CRFP hood and proposes specific design, complete manufacturing detail, experimental test on materials and the whole structure and numerical studies. There were investigated two different design concepts. The concepts are based in the first case (hood “A”) on an equivalent CFRP hood with similar geometry as those BMD design. The special design of composites that uses a light sandwich structure for the exterior side and changes the backside frame were adopted for the second case (hood “B”). Based on previous studies, the authors proposed new CFRP materials and stacking sequence for manufacturing the CFRP hoods, the proposed materials have not been analyzed before. Comparative studies between these concepts were performed by experimental and numerical methods.

The performed analyses firstly imply the measurement of elastic constants of the five different CFRP composites with the same architecture of the layers as those used for hood manufacturing. The manufacturing steps and full details regarding the fabrication process are delivered in the paper; a 3D scanning technique revealed the manufacturing dimensional accuracy with respect to initial CAD models. The deviations of the points can be observed in the backside of the frame for the CFRP hoods cases.

The mechanical behavior of the CFRP composite hoods was analyzed in terms of static stiffness and strain monitoring in three different points for lateral, longitudinal and torsional loading cases. Experimental set-ups include supporting conditions similar to the reality, load-displacements measuring sensors and strain gauges glued on the upper and lower surfaces of the hood. Numerical simulations with FE software of the investigated hoods revealed good agreement with the experimental data and validate both the experimental set-up and the developed numerical models.

In summary, the main article conclusion are as follows:

- The vacuum bag technology and autoclave curing process is the best procedure to produce very high quality CFRP parts, a fact demonstrated by microstructural analysis and highly dimensional accuracy of the manufactured hoods.
- A designed composite hood that copies the metal parts, by reproducing the same geometry shape and the reinforced ribs, brings the advantage of mass reduction with similar or higher stiffness, but limits the benefits that composites structures have.
- The first composite hood manufactured based on BMD is 2.54 times lighter than a similar steel hood and the improvement in terms of lateral stiffness for this composite hood about a similar steel hood is about 80%. Transversal stiffness is a few times, higher while the torsional stiffness has an increase of 62% as for a similar steel hood.
- The use of sandwich structures to achieve a high stiffness and light structure, a balanced stacking sequence of layers in order to respond to complex requests are the advantages of an FRP design concept that allows, in the case of a car hood replacement of the ribs and the reinforcements, an important additional mass reduction.
- The second hood concept is 22% lighter than the first one. In this case, the outer hood panel subjected to structural improvements by layer architecture offers a mass reduction about 53%. Lateral stiffness is also improved by 42%, while the transversal stiffness is significantly higher. The torsional load case revealed a smaller value, but not lower than for a similar steel hood.

The obtained results are consistent and will deliver support for future static and dynamic mechanical analyses of composite hoods both experimentally and by the finite element method In the future, improvements of the manufacturing technology for cost reduction (types of fabric, stacking sequence) will also be included, in addition to closer analysis of hinges area and backside frame. The main conclusions are already in implementation for other components, such vehicle doors, front and rear fenders and a trunk lid fabricated from CFRP composites.

## Figures and Tables

**Table 1:** The values of elastic constants obtained for 1A and 2A specimens.

Material	1A	2A
Test type	Modulus [MPa]	
Tension (E)	51,921	61,043
In plane shear (G)	24,962	29,633

**Table 2:** The values of elastic constants obtained for 1B, 2B and 3B specimens.

Material	1B	2B	3B
Test type	Modulus [MPa]		
Tension (E)	72,359	32,537	79,505
In plane shear (G)	35,821	12,051	35,433

**Table 3:** Measured stiffness of the composite hood “A”.

Load Case	Lateral	Transversal	Torsional
Applied force (N)	500	500	58
Displacement (mm)	1.73	1.18	0.73
Stiffness (N/mm)	288.7	425.7	79.5

**Table 4:** Experimental strain values measured in case of investigated load cases for hood “A”.

Load Case	Load Value [N]	Strain ε_SG1_ [μm/m]	Strain ε_SG2_ [μm/m]	Strain ε_SG3_ [μm/m]
Lateral	500	43.7	185.2	55.6
Transversal	500	1.02	2.3	5.9
Torsional	58	5.4	2.71	0.62

**Table 5:** Measured stiffness of the composite hood “B”.

Load Case	Lateral	Transversal	Torsional
Applied force (N)	230	504	206
Displacement (mm)	0.65	0.54	3.71
Stiffness (N/mm)	353.8	933.3	55.5

**Table 6:** Experimental strain values measured in case of investigated load cases for hood “B”.

Load Case	Load Value [N]	Strain ε_SG1_ [μm/m]	Strain ε_SG2_ [μm/m]	Strain ε_SG3_ [μm/m]
Lateral	230	73.8	53.3	72.8
Transversal	504	3.4	7.1	12.9
Torsional	206	18.6	27.8	15.6

**Table 7:** Stiffness of conventional steel hood (geometrically identical with “A” hood).

Material	Lateral Stiffness [N/mm]	Transversal Stiffness [N/mm]	Torsional Stiffness [N/mm]
Steel	162.3	75.6	58.6

**Table 8:** Computed displacements and stiffnesses of the composite hood “A”.

Load Case	Lateral	Transversal	Torsional
Applied force (N)	500	500	58
Computed displacement (mm)	1.71	1.36	0.61
Stiffness (N/mm)	292.4	367.64	95.1

**Table 9:** Computed strains on the outer and inner surface of the composite hood “A”.

Load Case	Load Value [N]	Strain ε_SG1_ [μm/m]	Strain ε_SG2_ [μm/m]	Strain ε_SG3_ [μm/m]
Lateral	500	48.8	180.8	53.9
Transversal	500	0.91	2.1	5.4
Torsional	58	5.2	2.6	0.70

**Table 10:** Material constants used in the simulation of honeycomb core.

Material		Honeycomb Core	
Young’s modulus [MPa]	E_1_ = 1	E_2_ = 1	E_3_ = 255
Shear modulus [MPa]	G_12_ = 10^−6^	G_31_ = 70	G_23_ = 37
Poisson’s ratio	ν_12_ = 0.49	ν_13_ = 0.001	ν_23_ = 0.001

**Table 11:** Elastic constants computed for reinforced frame and honeycomb structure.

Material	Reinforced Frame	Sandwich Structure
Young’s modulus [MPa]	52,085	6579
Shear modulus [MPa]	22,855	3256

**Table 12:** Computed displacements and stiffnesses of the composite hood “B”.

Load Case	Lateral	Transversal	Torsional
Applied force (N)	230	504	206
Computed displacement (mm)	0.55	0.56	3.53
Stiffness (N/mm)	418.2	900	58.4

**Table 13:** Computed strains on the outer and inner surface of the composite hood “B”.

Load Case	Load Value [N]	Strain ε_SG1_ [μm/m]	Strain ε_SG2_ [μm/m]	Strain ε_SG3_ [μm/m]
Lateral	230	85.8	52.9	82.9
Transversal	504	3.8	7.7	14.9
Torsional	206	19.2	30.4	17.3

## Data Availability

Not applicable.
